# Incidence and factors associated with intrauterine adhesions: An Australian population‐based longitudinal study

**DOI:** 10.1111/aogs.70352

**Published:** 2026-08-27

**Authors:** Chen Liang, Annette J. Dobson, Thierry Vancaillie, Richard Hockey, Gita D. Mishra

**Affiliations:** ^1^ Australian Women and Girls' Health Research Centre, School of Public Health The University of Queensland Brisbane Queensland Australia; ^2^ School of Women's and Children's Health University of New South Wales Sydney New South Wales Australia

**Keywords:** heavy periods, incidence, intrauterine adhesions, severe period pain, sociodemographic factors

## Abstract

**Introduction:**

Intrauterine adhesions (IUAs) describe a condition characterized by scar tissue that forms inside the uterus, which is associated with menstrual irregularities, infertility, and pregnancy loss. In the general population, the incidence of IUAs is largely unknown, as well as associated sociodemographic factors and other menstrual symptoms. This study aims to estimate the incidence of IUAs in the general population and explore the associated sociodemographic factors and menstrual symptoms.

**Material and Methods:**

Data from the Australian Longitudinal Study on Women's Health (ALSWH) was used. Women born in 1973–1978 were followed up from 1996 to 2021. The occurrence of IUAs was identified through hospital admissions and other health service records. The cumulative incidence and 5‐year incidence of reported IUAs with confidence intervals (CIs) were calculated using exact methods and the Poisson regression models, respectively. Log‐binomial models were used to assess the association between individual factors and reported IUAs.

**Results:**

Among the 13 133 women who were followed up from a median age of 20.7 years to 47.1 years, 2.7% (95% CI: 2.4–3.0%) had reported IUAs. The incidence rates of IUAs were below 5/10 000 person‐years for women aged < 30 and increased from 11.8/10 000 person‐years (95% CI: 9.4–14.7) to 15.7/10 000 person‐years (95% CI: 12.9–19.1) among women aged 31–35 to those aged 41–45, respectively. Women living in metropolitan areas (Risk Ratio [RR] = 1.47, 95% CI: 1.15–1.87), having private health insurance (RR = 3.29, 95% CI: 2.49–4.35), experiencing severe period pain sometimes or often (RR = 1.76, 95% CI: 1.32–2.34 or RR = 1.92, 95% CI: 1.37–2.68, respectively) or heavy periods sometimes or often (RR = 1.65, 95% CI: 1.25–2.18 or RR = 1.92, 95% CI: 1.39–2.65, respectively) were more likely to have recorded IUAs.

**Conclusions:**

In this national cohort, around one in 40 women had a report of IUAs, which was associated with better access to health services and menstrual symptoms. These findings clarify health issues related to IUAs and lay the groundwork for future studies.

## Graphical abstract

In an Australian national cohort, 2.7% women had reported intrauterine adhesions and the incidence rate increased gradually from women's early 30s to middle 40s, which was associated with better access to health services, severe period pain, and heavy periods.

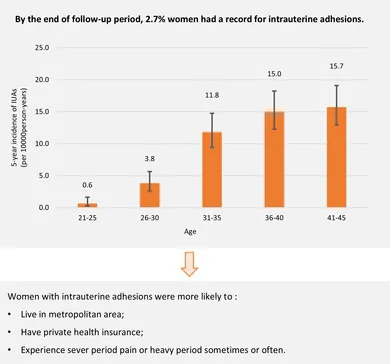

**Abbreviations**
ACHI: Australian Classification of Health Interventions
ALSWH: Australian Longitudinal Study on Women's Health
BMI: body‐mass index
CI: confidence interval
ICD: International Classification of Disease
IUAs: intrauterine adhesions
IUD: intrauterine device
MBS: medicare benefits schedule
RRs: risk ratios

**Key message**

The prevalence of intrauterine adhesions in the general population is largely unknown. In an Australian national cohort, 2.7% of women had reported intrauterine adhesions, which were associated with better access to health services, severe period pain, and heavy periods.

## 1 INTRODUCTION

Intrauterine adhesions (IUAs) is a condition characterized by a fibrotic repair process in place of the original scar‐free normal repair process for endometrial injury to form fibrous adhesive bands in the uterine cavity.^1^ Heinrich Fritsch first described IUAs in 1894, and then in 1948 Joseph G. Asherman recognized these adhesions and attributed the symptoms of amenorrhea, infertility, dysmenorrhea and recurrent pregnancy loss, and sequelae of complicated delivery or post‐abortion to this syndrome.^2^ IUAs can disturb endometrium shed, cervico‐utero‐tubal sperm transport, embryo migration and implantation, and placentation.^3^ Therefore, women with IUAs face an increased risk of menstrual irregularities (i.e., amenorrhea, hypomenorrhea, or dysmenorrhea), infertility, miscarriage, stillbirth, and preterm delivery.

It is difficult to estimate the exact prevalence of IUAs due to vague symptoms, lack of awareness about this condition, and uncertain causal factors. Additionally, the reported prevalence of IUAs varies depending on the study population, diagnostic methods, and geographic locations. In previous studies, the prevalence of IUAs has been estimated based on clinical presentations or surgeries, ranging from 1% to 2% after secondary amenorrhea to 45.5% after multiple myomectomies.^1^, ^4^ The prevalence of IUAs in the general population is largely unknown.

Several risk factors of IUAs have been identified, such as pregnancy loss, dilation and curettage, cesarean section, myomectomy, and infection.^4^, ^5^, ^6^, ^7^ These procedures or conditions may damage the uterine lining and subsequently increase the risk of adhesion between inner walls of the uterus.^8^ However, it is unclear whether the occurrence of IUAs differs by sociodemographic and lifestyle factors, which might account for different levels of awareness of IUAs and access to medical resources for IUAs diagnosis. In addition, little is known about the association of preexisting menstrual symptoms (i.e., irregular period, severe period pain, and heavy menstrual bleeding) with the diagnosis of IUAs. Those menstrual symptoms are usually accompanied by hormonal imbalance, abnormal endometrium shed, and bleeding disorders, which could contribute to the development of IUAs or the diagnosis of IUAs.

Therefore, this study used data from participants in the Australian Longitudinal Study on Women's Health (ALSWH) who were born in 1973–1978. The aims were to: (1) estimate the incidence of recorded IUAs over a 25‐year period (1996–2021); (2) explore the factors associated with the diagnosis of IUAs.

## 2 MATERIAL AND METHODS

### 2.1 Study population and participants

The ALSWH is a longitudinal population‐based cohort study, involving four age cohorts of Australian women (born in 1921–1926, 1946–1951, 1973–1978, and 1989–1995). In this study, data from the 1973 to 1978 cohort were used. Participants were randomly selected from the database of the national health insurance scheme, Medicare, which includes all Australian citizens and permanent residents. The cohort recruitment has been described previously.^9^ The survey was first completed in 1996 (when the women were aged 18–23 years), and then repeated every 3–4 years with the ninth survey completed in 2021 (participants aged 43–48 years). Linked data for the same period were obtained, including hospital admissions and Medicare Benefits Schedule (MBS) records (Table S1). Hospital admission data included diagnoses and procedures, while MBS data provided information on other health services subsidized by the Australian Government. Participants might not complete every survey, but their linked data have continued to be collected.

In this analysis, women were included if they consented to data linkage. None of these women had a diagnosis of IUAs at baseline, and information on covariates (e.g., geographic location, private health insurance status, education level, and smoking status) was provided in at least one survey.

### 2.2 Intrauterine adhesions

**FIGURE 1:**
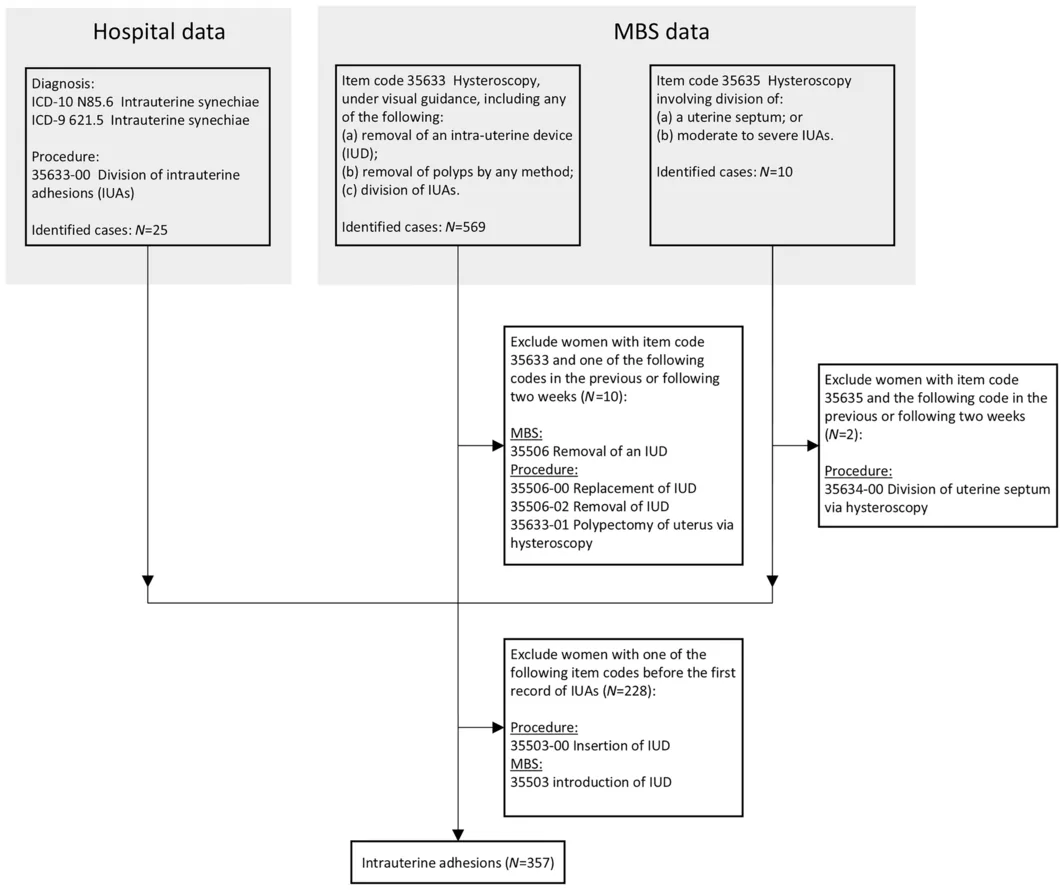
Identification of intrauterine adhesions.

The occurrence of IUAs was identified through hospital admission records using International Classification of Disease (ICD) codes and Australian Classification of Health Interventions (ACHI) codes, and from MBS item codes (Figure 1). In hospital admission data, hospital diagnosis (ICD‐10 N85.6 intrauterine synechiae; ICD‐9621.5 intrauterine synechiae) and procedures (ACHI 35633‐00 division of intrauterine adhesions) were used to identify women with IUAs. In MBS data, two items (35633 hysteroscopy for the removal of an intrauterine device [IUD], polyps, or division of minor IUAs; 35635 hysteroscopy for the division of uterine septum or moderate to severe IUAs) were used. Since these two MBS items could be used for other investigations or procedures (IUDs, polyps, and uterine septum procedures), women with other records indicating such conditions including a record of IUD insertion before the first record of these two MBS items were excluded because it was not possible to separate women having hysteroscopy for the removal of IUD from for the division of IUAs in the current coding system.

### 2.3 Menstrual symptoms

Menstrual symptoms were self‐reported in each survey. Participants were asked whether they had irregular periods, heavy periods, or severe period pain in the last 12 months with response options of never, rarely, sometimes, or often.

### 2.4 Sociodemographic and lifestyle factors

Information on geographic location (metropolitan area and other), private health insurance (yes and no), education level (less than high school, high school, certificate/diploma, and university or higher), body‐mass index (BMI; < 18.5, 18.5–24.9, 25.0–29.9, and ≥ 30.0 kg/m^2^), alcohol intake (nondrinker, rarely drinks, ≤ 2, and > 2 drinks per day), and smoking status (nonsmoker, past smoker, and current smoker) were collected at each survey. Due to the small number of women with records of IUAs in certain categories, education levels of less than high school, high school, and certificate/diploma were combined in the regression analysis, and alcohol intake categories of ≤ 2 and > 2 drinks per day were combined.

### 2.5 Statistical analysis

The cumulative incidence of IUAs was calculated by dividing the number of women with records indicative of IUAs by women without IUAs at cohort entrance. The cumulative incidence of IUAs was estimated every year, retaining participants in both the numerator and denominator after their first records of IUAs. Participants' age at the first record of IUAs was taken as age at IUA diagnosis. The cumulative incidence of IUAs was estimated as a binomial proportion, with confidence interval (CI) calculated using the Clopper–Pearson exact method.^10^ The 5‐year incidence rate of recorded IUAs was calculated. The follow‐up period from baseline, spanning ages 20 to 45, was divided into 5‐year intervals. The incidence rate for each interval was determined by dividing the number of new IUAs records by person‐year at risk during the corresponding interval. The incidence rate and 95% CIs were estimated using a Poisson regression model without any explanatory factors.^11^, ^12^, ^13^

Baseline sociodemographic and lifestyle factors were described according to the occurrence of recorded IUAs during the study period. The chi‐square test was used to compare the distribution among women with and without recorded IUAs. Log‐binomial models were used to estimate the risk ratios (RRs) and two‐sided 95% CIs for the association between individual factors identified in the survey and the first record of IUAs during the interval to the next survey. Data up to the record of IUAs or the last survey, whichever came first, were included in this analysis. Generalized estimating equation models, with an exchangeable correlation structure, were used to account for the repeated measures in the longitudinal data. The age effect was accounted for by including a fixed effect of age at survey completion in the model. First, each sociodemographic or lifestyle factor was examined separately, and then all the factors showing an association with recorded IUAs were included in a single model. After that, each menstrual symptom was assessed. All models were adjusted for sociodemographic and lifestyle factors associated with IUAs records.

In the above analysis, women who had insertion of an IUD before the first record of IUAs were excluded. To assess the impact of this limitation, a supplementary analysis including these women was conducted. In addition, to assess the influence of unmeasured confounding, E‐values were calculated, which indicated the minimum strength of association that an unmeasured confounder would need to have with both the exposure and the outcome, conditional on the measured covariates, to “explain away” an exposure–outcome association.^14^ All analyses were conducted using SAS version 9.4 (SAS Institute Inc., Cary, North Carolina, USA).

## 3 RESULTS

Overall, 13 133 women were included (Figure S1). They were recruited at a median (quartiles) age of 20.7 (19.5–22.0) and were followed up to a median (quartiles) age of 47.1 (45.9, 48.5). During the study period, 352 women were identified with records of IUAs at a median (quartiles) age of 38.8 (34.1–43.4).

**FIGURE 2:**
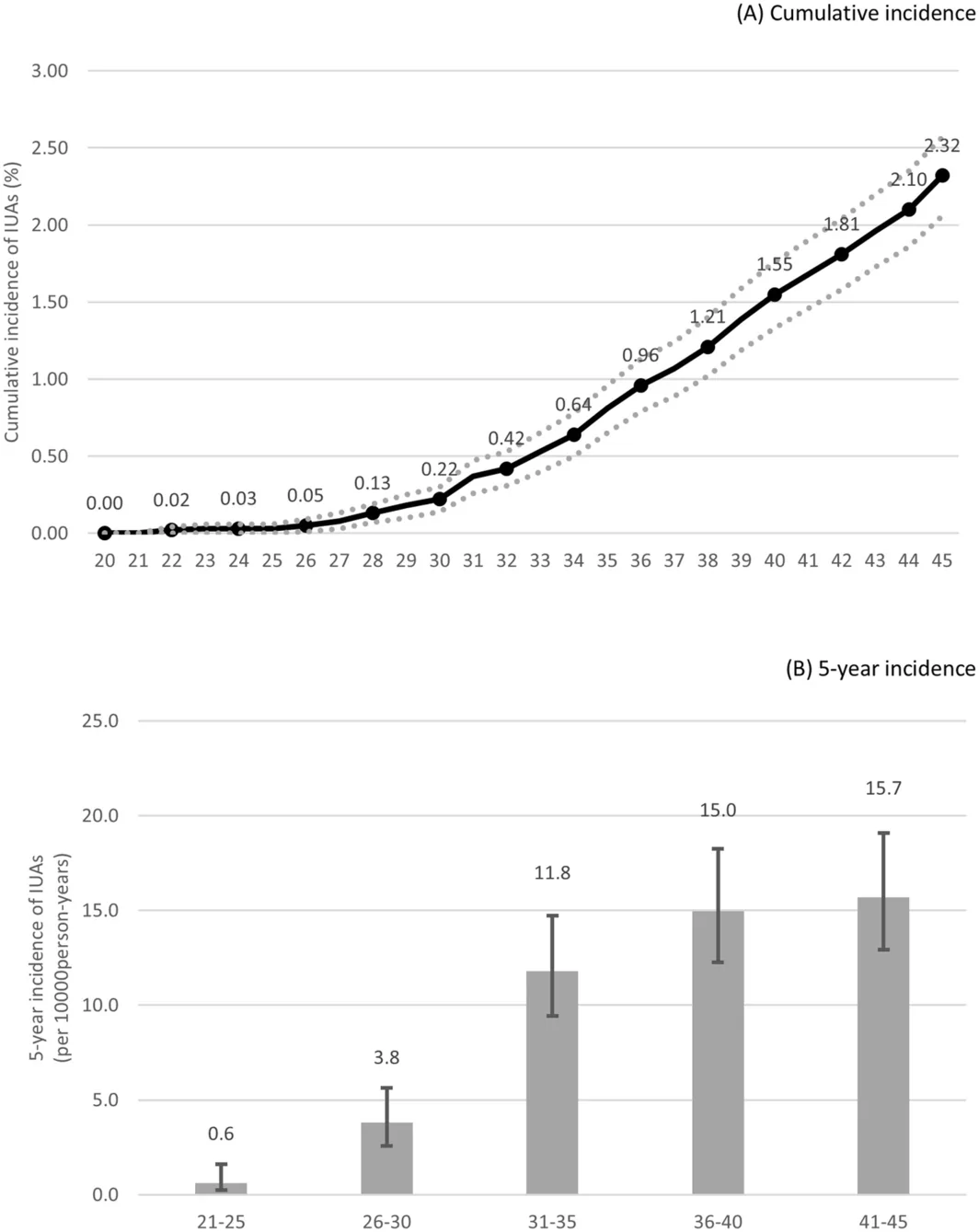
The cumulative incidence and 5‐year incidence of intrauterine adhesions. (A) Cumulative incidence: The dash lines indicate the 95% confidence intervals of cumulative incidence. Binomial proportion and Clopper–Pearson confidence limits were calculated. (B) 5‐year incidence: The error bars indicate the 95% confidence intervals of 5‐year incidence. Poisson regression models without any explanatory factors were used to estimate the incidence rate and 95% confidence intervals.

Among the included women, 2.7% (95% CI: 2.4–3.0%) had a record for IUAs by the end of follow‐up period. The cumulative incidence of recorded IUAs remained flat in women's 20s, and then gradually increased from 0.2% (95% CI: 0.1–0.3%) to 2.3% (95% CI: 2.1–2.6%) as their age rose from 30 to 45 years (Figure 2A). Similarly, the incidence rates of recorded IUAs were relatively low during women's 20s and then increased gradually from 11.8/10 000 person‐years (95% CI: 9.4–14.7) to 15.7/10 000 person‐years (95% CI: 12.9–19.1) when the women were aged 31–35 to when they were aged 41–45 (Figure 2B).

Baseline characteristics are presented in Table 1. Women living in metropolitan areas, having private health insurance, having a higher level of education, and never smoking at baseline were more likely to have a record of IUAs.

**TABLE 1:** Baseline sociodemographic and lifestyle factors between women with and without recorded intrauterine adhesions during the follow‐up period.

		Intrauterine adhesions		*p*
		Never	Ever	
Geographic location	Metropolitan area	7012 (96.9)	221 (3.1)	0.004
	Other	5742 (97.8)	131 (2.2)	
Private health insurance	Yes	3796 (96.6)	132 (3.4)	0.001
	No	8860 (97.7)	213 (2.4)	
Education	Less than high school	2237 (98.4)	36 (1.6)	0.001
	High school	6804 (97.1)	200 (2.9)	
	Certificate/diploma	2281 (97.4)	62 (2.7)	
	University or higher	1405 (96.4)	52 (3.6)	
BMI	≤ 18.4	1080 (97.1)	32 (2.9)	0.991
	18.5–24.9	7519 (97.2)	217 (2.8)	
	25.0–29.9	1794 (97.1)	53 (2.9)	
	≥ 30.0	732 (97.3)	20 (2.7)	
Alcohol intake	Nondrinker	1130 (97.7)	27 (2.3)	0.543
	Rarely drink	4362 (97.4)	115 (2.6)	
	≤ 2 drinks per day	6470 (97.1)	191 (2.9)	
	> 2 drinks per day	695 (97.8)	16 (2.3)	
Smoking status	Nonsmoker	6366 (96.7)	220 (3.3)	< 0.001
	Past smoker	1906 (98.1)	37 (1.9)	
	Current smoker	4066 (98.0)	84 (2.0)	

*Note*: There were 352 women with records of intrauterine adhesions during the follow‐up period, and 12 718 women were without records of intrauterine adhesions. Chi‐square test was used to compare the distribution among women with and without intrauterine adhesions.

Association between sociodemographic or lifestyle factors and a record of IUAs was assessed. In univariate models, women living in metropolitan areas and having private health insurance were more likely to have a record of IUAs, while women with lower education levels and smoking (past or current) were less likely to have IUAs recorded (Table 2). After including these four factors in the same model, only the associations with living in metropolitan areas (RR = 1.47, 95% CI: 1.15–1.87) and having private health insurance (RR = 3.29, 95% CI: 2.49–4.35) persisted (Table 2).

**TABLE 2:** Association of sociodemographic, lifestyle factors, and menstrual symptoms in the previous survey with the first record of intrauterine adhesions in the interval up to the next survey.

		Model 1	Model 2	Model 3
Geographic location	Metropolitan area	1.85 (1.46, 2.36)	1.47 (1.15, 1.87)	1.51 (1.18, 1.93)
	Other	Ref.	Ref.	Ref.
Private health insurance	Yes	3.81 (2.93, 4.94)	3.29 (2.49, 4.35)	3.64 (2.79, 4.76)
	No	Ref.	Ref.	Ref.
Education level	Less than university	0.53 (0.43, 0.67)	0.86 (0.68, 1.08)	‐
	University or higher	Ref.	Ref.	‐
BMI	≤ 18.4	1.17 (0.73, 1.88)	‐	‐
	18.5–24.9	Ref.	‐	‐
	25.0–29.9	0.79 (0.59, 1.06)	‐	‐
	≥ 30.0	0.91 (0.67, 1.23)	‐	‐
Alcohol intake	Non‐drink	Ref.	‐	‐
	Rarely drink	1.02 (0.67, 1.55)	‐	‐
	Other	1.36 (0.93, 1.99)	‐	‐
Smoking status	Never	Ref.	Ref.	‐
	Past smoker	0.73 (0.55, 0.97)	0.81 (0.61, 1.08)	‐
	Current smoker	0.51 (0.37, 0.70)	0.75 (0.54, 1.05)	‐
Irregular period	Never	Ref.	‐	Ref.
	Rarely	1.30 (0.97, 1.75)	‐	1.37 (1.02, 1.83)
	Sometimes	1.19 (0.86, 1.65)	‐	1.26 (0.91, 1.75)
	Often	1.10 (0.75, 1.61)	‐	1.22 (0.83, 1.78)
Severe period pain	Never	Ref.	‐	Ref.
	Rarely	1.33 (0.99, 1.77)	‐	1.37 (1.03, 1.83)
	Sometimes	1.59 (1.20, 2.12)	‐	1.76 (1.32, 2.34)
	Often	1.64 (1.18, 2.30)	‐	1.92 (1.37, 2.68)
Heavy periods	Never	Ref.	‐	Ref.
	Rarely	1.05 (0.76, 1.45)	‐	1.09 (0.79, 1.51)
	Sometimes	1.57 (1.18, 2.07)	‐	1.65 (1.25, 2.18)
	Often	1.77 (1.28, 2.44)	‐	1.92 (1.39, 2.65)

*Note*: All data are presented as risk ratios and two‐sided 95% confidence intervals. Log‐binomial models were fitted. Generalized estimating equation models, with an exchangeable correlation structure, accounted for the repeated measures in the longitudinal data. Aging was taken into account by including a fixed effect of age at survey completion. Model 1 included single sociodemographic factor, lifestyle factor, or menstrual symptom. Model 2 included all the sociodemographic and lifestyle factors which were associated with the diagnosis of intrauterine adhesions in model 1 (i.e., geographic location, private health insurance, education level, and smoking status). Model 3 included single menstrual symptom and all the sociodemographic and lifestyle factors which were associated with the diagnosis of intrauterine adhesions in model 2 (i.e., geographic location and private health insurance). The effect sizes of geographic location and private health insurance in model 3 came from the model with irregular period. Their effect sizes in model 3 with severe period pain or heavy periods were (RR = 1.51, 95% CI: 1.18–1.93 and RR = 3.77, 95% CI: 2.89–4.91) or (RR = 1.52, 95% CI: 1.1–1.94 and RR = 3.67, 95% CI: 2.81–4.79).

Then the associations between menstrual symptoms and a record of IUAs were assessed. Women who experienced severe period pain sometimes or often were more likely to have a record of IUAs (RR = 1.76, 95% CI: 1.32–2.34 and RR = 1.92, 95% CI: 1.37–2.68, respectively; Table 2). Similar associations were observed among women having heavy periods sometimes or often (RR = 1.65, 95% CI: 1.25–2.18 and RR = 1.92, 95% CI: 1.39–2.65 respectively; Table 2).

In the supplementary analysis, the cumulative incidence of recorded IUAs increased to 4.34% (95%: 4.00–4.69%) when women with a history of IUD insertion were included. The cumulative incidence and 5‐year incidence rates showed similar patterns with the main analysis: low in women's 20s and increased gradually after age 30 (Figure S2). *E*‐values showed the robustness of the observed association to unmeasured confounding. For the association with private health insurance to be explained by an unmeasured confounder, it would need to have a risk ratio of at least 6.74 with both the exposure (private health insurance) and the outcome (an IUA record) (Table S2). Comparatively, weaker but still strong confounder associations would be needed to explain the observed associations for geographic location (RR = 2.38), severe period pain sometimes or often (RR = 2.91 or RR = 3.24), and heavy period sometimes or often (RR = 2.68 or RR = 3.25) (Table S2).

## 4 DISCUSSION

In this longitudinal cohort study, 2.7% of participants had a record suggestive of IUAs. The incidence rate of recorded IUAs was relatively low in women's 20s, and then gradually increased from their early 30s to middle 40s. Women were more likely to have IUAs recorded if they lived in a metropolitan area, had private health insurance, experienced severe period pain or heavy periods sometimes or often.

Patients with IUAs are commonly diagnosed after they have experienced menstrual symptoms (e.g., menstrual irregularity, decreased menstrual flow, or severe period pain), infertility, or pregnancy loss, and seek medical help. Most IUAs diagnosis would occur during the reproductive life stages. In this study, women were followed up from their early 20s to middle 40s, which enabled researchers to identify records of IUAs across much of the reproductive lifespan and describe how the incidence changed with women's age. As far as we know, this is the first study to report the incidence of IUAs by age in the general population. This information helps researchers understand how the risk of developing IUAs changes across life course. In addition, hospital and other health service data were used to identify women with records suggestive of IUAs. The hospital data included hospital diagnoses and procedures, while the MBS data covered both in‐hospital and out‐of‐hospital services. These two data sources allowed researchers to identify women with IUAs records treated as inpatients or outpatients. Multiple linkage data sources improved case ascertainment and reduced misclassification bias.

However, several limitations should be mentioned. Compared to the Australian Census 2011, women in the ALSWH cohort born in 1973–1978 were slightly more likely to be better educated and employed than women of the same age in the general population, and these differences have increased since then. It was important to consider the effect of education and employment on having a record of IUAs. Having a higher education level and being in paid employment are generally associated with better health, which might have led to underestimation of the incidence of IUAs.^15^, ^16^ However, women with a higher education level and better economic status may also have better access to medical resources, which can result in a higher chance of being diagnosed with IUAs, leading to overestimation of IUAs incidence. Additionally, this study could only count women with a record of diagnosis or treatment history of IUAs. Therefore, women with asymptomatic IUAs might be undiagnosed, and this might lead to underestimation of the incidence. Furthermore, in the current coding system of hospital data, there are specific ICD or procedure codes for IUAs. However, the MBS items for IUAs can be used for other indications. Although several criteria were applied to identify women who underwent these procedures for IUAs, some women might still have been misclassified. In supplementary analysis, when women with any of the hospital ICD codes or MBS items were included, the cumulative incidence of IUAs increased to 4.34%. Finally, unmeasured confounding might exist in the observed associations with geographic location, private health insurance, severe period pain, and heavy periods. But E‐values indicated the strong associations (RRs ranging from 2.38 to 6.74) that any unmeasured confounder would need to have with one of these four exposure factors and IUAs to fully explain the observed associations.

Previous studies have shown that the prevalence of IUAs varies across different settings of clinical presentations and surgical procedures.^1^ Examples of the incidence of IUAs have been reported as follows: 1–2% among women with secondary amenorrhea, 1–4% following cesarean section, 6–7% in women with infertility, 10–30% after pregnancy termination, 10–31% following miscarriage, 15–40% after curettage, 19–27% in women with postpartum hemorrhage 22–32% following uterine septum resection, and 31–46% after myoma resection.^1^, ^6^, ^17^, ^18^, ^19^, ^20^, ^21^, ^22^, ^23^, ^24^, ^25^ To our knowledge, no previous studies have assessed the occurrence of IUAs among a general population. The present study showed that the cumulative incidence of IUAs was 2.7% (95% CI: 2.4–3.0%) in a nationally representative sample of Australian women.

In addition, the present study explored the associations between sociodemographic factors, lifestyle factors, menstrual symptoms, and recorded IUAs. Previous studies mainly focused on the role of clinical factors (i.e., gravidity, miscarriage, pregnancy termination, infection) and surgical procedures (dilation curettage, myomectomy, polypectomy, pelvic radiation) in the development of IUAs.^6^, ^7^ To the best of our knowledge, only one previous study explored the association between menstrual disorders and IUAs. Westendorf et al. enrolled 50 women undergoing secondary removal of placental remnants after incomplete abortion, and found the following associations with IUAs: any menstrual disorders (RR = 12, 95% CI: 1.7–58), amenorrhea (RR = 15, 95% CI: 2.1–109), dysmenorrhea (RR = 15, 95% CI: 1.9–122), or hypomenorrhea (RR = 6.9, 95% CI: 0.81–59).^24^ However, study limitations, such as an unrepresentative, small sample, and single‐time data collection, limited their findings and the generalizability to the broader population. The present study enrolled over 13 000 women from the general population and collected information on menstrual symptoms every 3 years and showed that women experiencing severe period pain or heavy periods sometimes or often were more likely to have IUAs recorded. Uterine surgeries, such as cesarean sections, can lead to severe period pain and heavy periods through myometrial hypertrophy, as well as IUAs from uterine trauma.^26^ Additionally, adhesions can block menstrual flow and cause severe period cramps, and the treatment of heavy periods (e.g., endometrial resection and ablation) may contribute to the development of IUAs.^27^, ^28^ Further, women with severe period pain or heavy period may be more likely to seek medical care. Their greater use of healthcare may increase diagnostic opportunities, leading to a higher probability of IUA diagnosis independent of underlying biological risk.

In this study, women living in metropolitan areas or having private health insurance were more likely to have a record of IUAs. Geographic differences may be due to greater awareness of reproductive complications and better access to healthcare services.^29^ Rural populations may experience lower access to healthcare compared to urban populations, while greater private health insurance coverage also increases patients' access to healthcare facilities and promotes earlier detection of disease.^30^, ^31^ Therefore, differences in IUAs records are likely to be associated with inequitable access to diagnostic services.

Additionally, the incidence of reported IUAs increased with women's age, especially from their early 30s to middle 40s. In Australia, around 64% of the women who gave birth in 2022 were over 30 years old.^32^ Given that infertility and pregnancy loss are each associated with both IUAs and older age, women over 30 years with IUAs are likely to be at greater risk of pregnancy difficulties.^3^

Further, women experiencing severe period pain or heavy periods were more likely to have IUAs recorded. It is unclear whether the observed association reflects an underlying biological mechanism or increased healthcare use and diagnostic ascertainment among women with these menstrual symptoms. Future studies on potential biological mechanisms (e.g., hormonal level change, uterine structure, uterine tissue ischemia, inflammation, and endometrial regeneration) and healthcare use are needed to shed more light on this issue.^1^, ^4^ In the present study, women in metropolitan areas and those with private health insurance were more likely to report menstrual symptoms (Figures S3, S4).^33^ Therefore, the association between recorded IUAs and menstrual symptoms increased after adjustment for area of residence and insurance status.

Information on the incidence of IUAs can improve understanding of health issues facing women, including the need to improve equity of access to services and preventive or treatment programs.

## 5 CONCLUSION

In a national cohort of Australian women at reproductive age, around one in 40 women had a record of IUAs. The incidence rate was relatively low in women's 20s and then increased gradually from early 30s to middle 40s. Women with records of IUAs were more likely to live in metropolitan areas, have private health insurance, and experience severe period pain or heavy periods sometimes or often. Future studies are needed to investigate whether and how these observed associations are driven by underlying biological pathways or by differences in healthcare‐seeking behavior and diagnostic opportunities. Establishing the incidence and describing the factors associated with recorded IUAs lays the groundwork for future studies on potential causes, prevention measures, and intervention strategies. This information could also help to allocate health resources more equitably.

## AUTHOR CONTRIBUTIONS

CL: Conceptualization, formal analysis, investigation, methodology, resources, writing—original draft, and writing—review and editing. AJD: Conceptualization, methodology, supervision, and writing—review and editing. TV: Writing—review and editing. RH: Data curation. GDM: Conceptualization, funding acquisition, methodology, resources, supervision, and writing—review and editing.

## FUNDING INFORMATION

This study was funded by NHMRC Centre for Research Excellence (APP1153420), and NHRMC Investigator grant (APP2009577). The funding source had no role in the study design, data collection, analysis, interpretation, and publication of this study.

## CONFLICT OF INTEREST STATEMENT

The authors declare no conflicts of interest.

## ETHICS STATEMENT

Ethics approval for ALSWH was obtained from the Human Research Ethics Committee (HREC) of the University of Newcastle (H‐076‐0795 and H‐2011‐0371) on July 26, 1995 and Queensland (2004/HE000224 and 2012/HE000132) on January 31, 2012. Ethical approval for linkage data was additionally obtained from the Australian Institute of Health and Welfare HREC (No. EC2020/03/1115) and appropriate HREC in each state. All participants consented to join the study and to the linkage of their health records. They are free to withdraw or suspend their participation at any time with no need to provide a reason.

## Supporting information


**Figure S1:** Flowchart. IUAs, intrauterine adhesions; IUD, intrauterine device.
**Figure S2:** The cumulative incidence and 5‐year incidence of intrauterine adhesions after including women who had insertion of IUD before the first record of IUAs. (A) Cumulative incidence: the dash lines indicate the 95% confidence intervals of prevalence. Binomial proportion and Clopper–Pearson confidence limits were calculated. (B) 5‐year incidence: the error bars indicate the 95% confidence intervals of incidence. Poisson regression models without any explanatory factors were used to estimate the incidence rate and 95% confidence intervals.
**Figure S3:** The percentage of menstrual symptoms by areas of residence. The figure shows the percentage of women reporting certain menstrual symptom sometimes or often.
**Figure S4:** The percentage of menstrual symptoms by private health insurance status. The figure shows the percentage of women reporting certain menstrual symptom sometimes or often.
**Table S1:** Linkage data sources.
**Table S2:** *E*‐value for risk ratios between sociodemographic factors, menstrual symptoms, and a record of intrauterine adhesions.

## Data Availability

The ALSWH data are available on request to the ALSWH Data Access Committee. The guidelines on how to apply for access to the data are at https://alswh.org.au/for‐data‐users/applying‐for‐data/.
