# Supplementary material for: Incidence and factors associated with intrauterine adhesions: An Australian population‐based longitudinal study
Source: Acta Obstet Gynecol Scand. 2026 Aug 27:10.1111/aogs.70352. Online ahead of print. doi: 10.1111/aogs.70352 (PMC13522671; doi:10.1111/aogs.70352)
Supplement: Supplementary file 1 [file AOGS-9999-0-s001.docx]

**Incidence and factors associated with intrauterine adhesions: an Australian population-based longitudinal cohort study**

[Table S1. Linkage data sources	2](#_Toc221699415)

[Table S2. E-value for risk ratios between sociodemographic factors, menstrual symptoms and a record of intrauterine adhesions	3](#_Toc221699416)

[Figure S1. Flow chart	4](#_Toc221699417)

[Figure S2. The cumulative incidence and 5-year incidence of intrauterine adhesions after including women who had insertion of IUD before the first record of IUAs	5](#_Toc221699418)

[Figure S3. The percentage of menstrual symptoms by areas of residence	6](#_Toc221699419)

[Figure S4. The percentage of menstrual symptoms by private health insurance status	7](#_Toc221699420)

# Table S1. Linkage data sources

|  | State | From | To | Provider |
| --- | --- | --- | --- | --- |
| Hospital admission data | Australian Capital Territory | July 2004 | June 2020 | The Centre for Health Record Linkage (CHeReL), NSW Ministry of Health and ACT Health |
|  | New South Wales | June 2001 | March 2021 |  |
|  | Queensland | February 2002 | September 2022 | Queensland Health and the Statistical Analysis and Linkage Unit (Queensland Health) |
|  | South Australia | June 2000 | June 2020 | South Australia North Territory DataLink, South Australia Health, and Northern Territory Department of Health |
|  | Northern Territory | June 2000 | June 2019 |  |
|  | Tasmania | January 2007 | December 2019 | The Department of Health Tasmania, and the Tasmanian Data Linkage Unit |
|  | Victoria | January 2000 | December 2020 | Victorian Department of Health and the Centre for Victorian Data Linkage (Victorian Department of Health) |
|  | West Australia | January 1970 | August 2021 | The Department of Health Western Australia |
| MBS data | All state | February 1984 | December 2020 | The Australian Government Department of Health and Aged Care |

# Table S2. E-value for risk ratios between sociodemographic factors, menstrual symptoms and a record of intrauterine adhesions

| Factors | Comparison | RR (95%CI) | E-value |
| --- | --- | --- | --- |
| Geographic location | Metropolitan area vs Other | 1.51 (1.18, 1.93) | 2.38 (1.64, 3.27) |
| Private health insurance | Yes vs No | 3.64 (2.79, 4.76) | 6.74 (5.02, 9.00) |
| Severe period pain | Rarely vs Never | 1.37 (1.03, 1.83) | 2.08 (1.21, 3.06) |
|  | Sometimes vs Never | 1.76 (1.32, 2.34) | 2.91 (1.97, 4.11) |
|  | Often vs Never | 1.92 (1.37, 2.68) | 3.24 (2.08, 4.80) |
| Heavey periods | Rarely vs Never | 1.09 (0.79, 1.51) | 1.40 (1.00, 2.88) |
|  | Sometimes vs Never | 1.65 (1.25, 2.18) | 2.68 (1.81, 3.78) |
|  | Often vs Never | 1.92 (1.39, 2.65) | 3.25 (2.13, 4.74) |

# Figure S1. Flow chart

IUD: intrauterine device. IUAs: intrauterine adhesions.

# Figure S2. The cumulative incidence and 5-year incidence of intrauterine adhesions after including women who had insertion of IUD before the first record of IUAs

(A) Cumulative incidence: the dash lines indicate the 95% confidence intervals of prevalence. Binomial proportion and Clopper-Pearson confidence limits were calculated. (B) 5-year incidence: the error bars indicate the 95% confidence intervals of incidence. Poisson regression models without any explanatory factors were used to estimate the incidence rate and 95% confidence intervals.

# Figure S3. The percentage of menstrual symptoms by areas of residence

(A) irregular period

|  | metropolitan | other |
| --- | --- | --- |
| 1 | 25.17 | 23.1 |
| 2 | 22.3 | 20.94 |
| 3 | 21.49 | 21.19 |
| 4 | 21.84 | 22.17 |
| 5 | 21.11 | 21.08 |
| 6 | 22.6 | 22.79 |
| 7 | 21.79 | 21.89 |
| 8 | 21.14 | 21.41 |
| 9 | 25.4 | 25.14 |

(B) Severe period pain

|  | metropolitan | other |
| --- | --- | --- |
| 1 | 39.83 | 40.06 |
| 2 | 31.08 | 29.6 |
| 3 | 28.52 | 28.18 |
| 4 | 27.37 | 28.05 |
| 5 | 25.7 | 27.11 |
| 6 | 26.79 | 28.28 |
| 7 | 27.81 | 29.47 |
| 8 | 26.65 | 28.77 |
| 9 | 26.41 | 28.49 |

(C) Heavy periods

|  | metropolitan | other |
| --- | --- | --- |
| 1 | 27.25 | 27.95 |
| 2 | 20.42 | 20.38 |
| 3 | 20.01 | 22.06 |
| 4 | 22.27 | 25.13 |
| 5 | 22.56 | 25.42 |
| 6 | 27.49 | 29.53 |
| 7 | 29.26 | 30.35 |
| 8 | 29.82 | 30.07 |
| 9 | 30.58 | 30.75 |

The figure shows the percentage of women reporting certain menstrual symptom sometimes or often.

# Figure S4. The percentage of menstrual symptoms by private health insurance status

(A) Irregular period

|  | with | without |
| --- | --- | --- |
| 1 | 23.89 | 24.46 |
| 2 | 19.89 | 22.39 |
| 3 | 18.8 | 22.79 |
| 4 | 20.57 | 23.01 |
| 5 | 18.94 | 23.16 |
| 6 | 21.15 | 24.29 |
| 7 | 19.8 | 24.09 |
| 8 | 19.09 | 23.67 |
| 9 | 24.29 | 26.41 |

(B) Severe period pain

|  | with | without |
| --- | --- | --- |
| 1 | 39.14 | 40.24 |
| 2 | 28.4 | 31.19 |
| 3 | 25.07 | 30.17 |
| 4 | 23.9 | 30.41 |
| 5 | 21.87 | 30.49 |
| 6 | 23.56 | 31.46 |
| 7 | 25.09 | 32.27 |
| 8 | 23.82 | 31.67 |
| 9 | 23.53 | 31.49 |

(C) Heavy periods

|  | with | without |
| --- | --- | --- |
| 1 | 25.87 | 28.38 |
| 2 | 18.54 | 21.18 |
| 3 | 17.93 | 22.56 |
| 4 | 20.72 | 25.51 |
| 5 | 20.84 | 26.57 |
| 6 | 27.02 | 29.81 |
| 7 | 28.78 | 30.82 |
| 8 | 29.18 | 30.8 |
| 9 | 29.91 | 31.53 |

The figure shows the percentage of women reporting certain menstrual symptom sometimes or often.
